# Infectious pancreatic necrosis virus enters CHSE-214 cells via macropinocytosis

**DOI:** 10.1038/s41598-017-03036-w

**Published:** 2017-06-08

**Authors:** Jorge Levican, Camila Miranda-Cárdenas, Ricardo Soto-Rifo, Francisco Aguayo, Aldo Gaggero, Oscar León

**Affiliations:** Programa de Virología, Instituto de Ciencias Biomédicas, Facultad de Medicina, Universidad de Chile, Santiago, Chile

## Abstract

Infectious pancreatic necrosis virus (IPNV) is a non-enveloped virus belonging to the *Birnaviridae* family. IPNV produces an acute disease in salmon fingerlings, with high mortality rates and persistent infection in survivors. Although there are reports of IPNV binding to various cells, the viral receptor and entry pathways remain unknown. The aim of this study was to determine the endocytic pathway that allows for IPNV entry. We observed that IPNV stimulated fluid uptake and virus particles co-localysed with the uptake marker dextran in intracellular compartments, suggesting a role for macropinocytosis in viral entry. Consistent with this idea, viral infection was significantly reduced when the Na+/H+ exchanger NHE1 was inhibited with 5-(N-Ethyl-N-isopropyl) amiloride (EIPA). Neither chlorpromazine nor filipin complex I affected IPNV infection. To examine the role of macropinocytosis regulators, additional inhibitors were tested. Inhibitors of the EGFR pathway and the effectors Pak1, Rac1 and PKC reduced viral infection. Together, our results indicate that IPNV is mainly internalized into CHSE-214 cells by macropinocytosis.

## Introduction

Infectious pancreatic necrosis virus (IPNV), a dsRNA virus belonging to the *Birnaviridae* family, is the etiological agent of infectious pancreatic necrosis, which affects several salmonid species. This disease has great economic impact in all salmon-farming countries. IPNV produces high mortality in young salmonids^1^, and in later stages of salmon development, the infection becomes persistent and difficult to eradicate^2^.

The IPNV genome is composed of two dsRNA segments, designated A and B. Segment A encodes a 107-kDa precursor polyprotein that gives rise to the structural proteins VP2 and VP3 and the nonstructural protein VP4^3^. VP5, a nonstructural protein, is encoded in a small open reading frame at the 5′ end of segment A^4^. Segment B encodes VP1, a RNA-dependent RNA polymerase. The full viral replication cycle takes about 24 h at 15 °C. Transcription intermediates are detected between 2 and 4 hours post infection (hpi)^5^, and viral proteins are produced between 3 and 14 hpi^6^. A ribonucleoprotein complex containing a full-length negative strand has been proposed as an intermediate for genome replication^7^. However, little is known about the early events of IPNV replication such as the nature of the IPNV receptor or the pathways used for cell entry. A 220-kDa protein from the CHSE-214 cell line, derived from a Chinook Salmon (Oncorhynchus tshawytscha) embryo, has been found to be associated with IPNV binding^8^; however, no further identifying data are available.

Although clathrin coat-resembling vesicles have been observed in infected cells^9^, other studies show that IPNV does not require low endosomal pH for endocytosis^10^. Thus, a systematic study of the endocytosis pathway involved in IPNV entry during infection would be useful.

Virus entry pathways include clathrin-mediated endocytosis (CME), lipid raft-mediated endocytosis, membrane raft-mediated endocytosis and macropinocytosis^11–13^. To identify the cellular pathway that allows for IPNV entry into CHSE-214 cells, we took advantage of several well-characterized inhibitors of known components of these endocytosis mechanisms and analyzed their impact on viral internalisation.

In this communication, we examined the roles of macropinocytosis, CME and lipid raft-mediated endocytosis in IPNV entry into CHSE-214 cells. Our results show that IPNV stimulates fluid uptake by CHSE-214 cells and that inhibition of the Na^+^/H^+^ pump with 5-(N-Ethyl-N-isopropyl) amiloride (EIPA) reduces IPNV entry and infection Macropinocytosis inhibitors that do not affect CME or lipid raft/caveolin-mediated endocytosis also impede viral infection, suggesting that macropinocytosis is a major pathway for IPNV entry into CHSE-214 cells.

## Results

### IPNV infection of CHSE-214 cells

**Figure 1:**
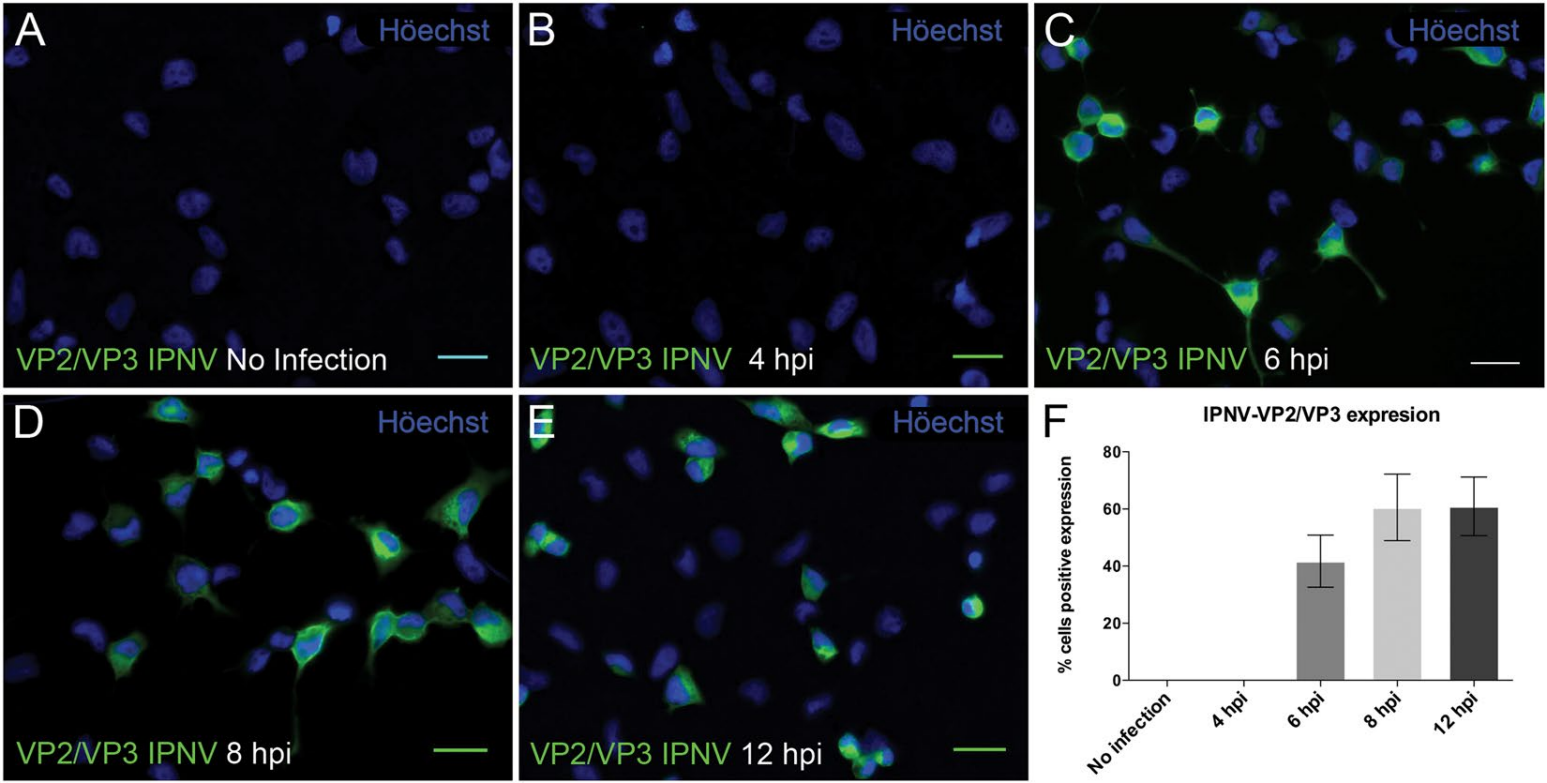
Timecourse of IPNV infection of CHSE-214 cells. VP2/VP3 expression was determined by immunofluorescence after 4 (**B**), 6 (**C**), 8 (**D**) and 12 (**E**) h post infection (hpi) in CHSE-214 cells. Cells were propagated at 60–70% confluence in 24-well plates with round glass coverslips. IPNV was inoculated at a MOI of 1 for 1 h. After 4, 6, 8 and 12 h of incubation at 20 °C, cells were processed by indirect immunofluorescence (IFI) using mouse oligoclonal anti-VP2/VP3 IPNV antibody and Alexa 488-conjugated donkey anti-mouse antibody. Representative images, recorded with an Olympus Spinning Disk IX81 microscope, are shown (bar scale = 20 μm). At each incubation time, 250 cells were counted. Number of VP2/VP3-expressing cells is shown as a percentage, normalized to mock cells (**F**).

Viral infection kinetics were determined by observing the synthesis of structural proteins VP2 and VP3 with immunofluorescence, using a mouse oligoclonal antibody against these proteins. As shown in Fig. 1, VP2- and VP3-containing polypeptides were detected 6 hpi (Fig. 1C). This observation is consistent with previous studies on virus-specific protein using pulse-labeling of infected cultures with [^35^S]-methionine, which reported that the primary gene products were produced 3–14 hpi, peaking at 6–9 hpi^6^. The percentage of infected cells plateaued at 8–12 hpi (Fig. 1D–E). Figure 1F shows the percentage of cells expressing VP2 and VP3 as an indicator of the extent of infection. This assay was used in the following experiments to examine the impact of inhibitors of macropinocytosis, CME and lipid raft-/caveolin-mediated endocytosis in IPNV entry.

### IPNV infection is not blocked by chlorpromazine

**Figure 2:**
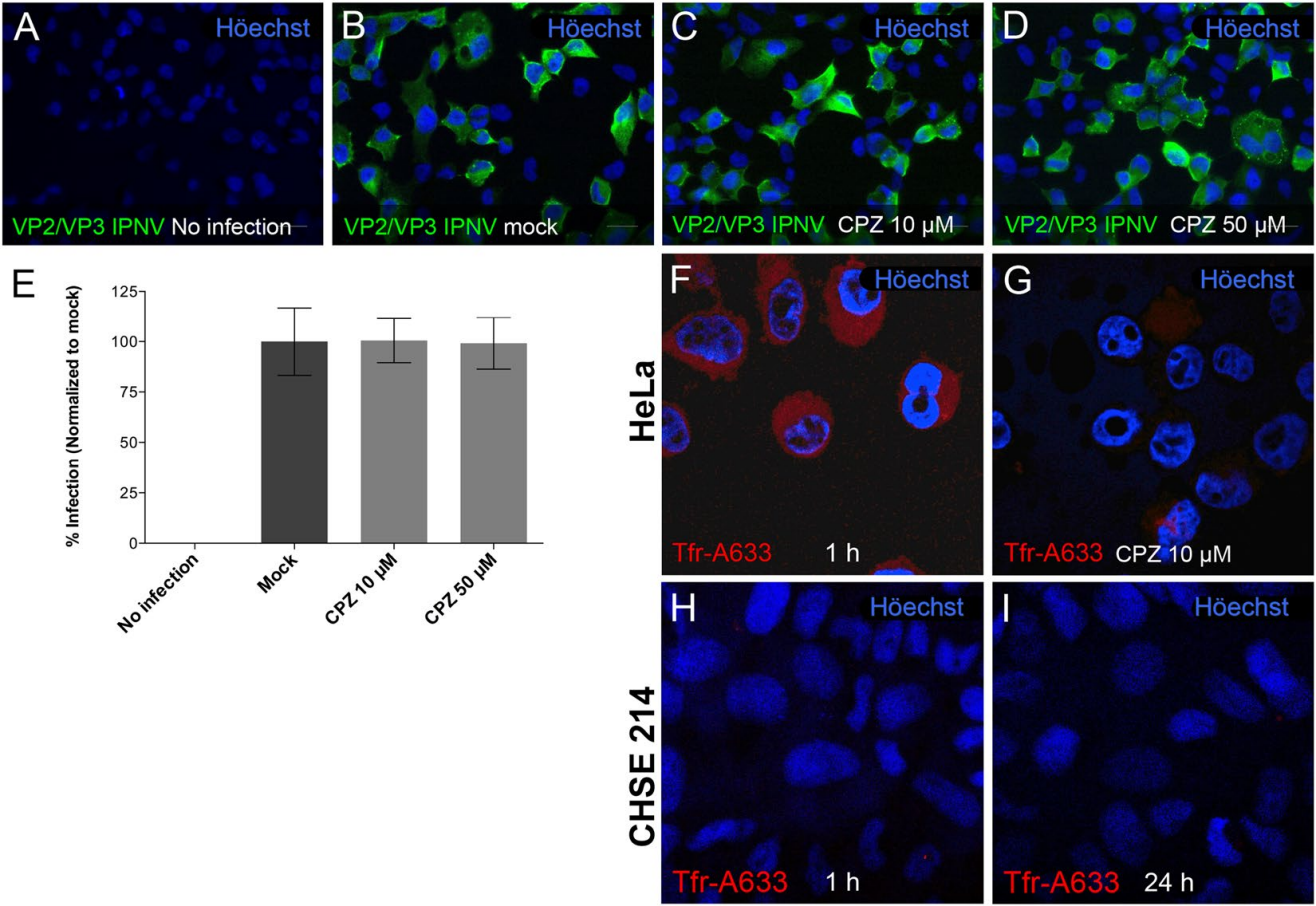
Chlorpromazine does not affect IPNV infection in CHSE-214 cells. CHSE-214 cells were propagated to 70–80% confluence in 24-well plates with round glass coverslips. IPNV was inoculated for 1 h in the presence of 0 (**B**), 10 (**C**) or 50 (**D**) μM CPZ. After 8 h of incubation at 20 °C, cells were processed by indirect immunofluorescence (IFI) for IPNV VP2/VP3 and images were recorded using a Olympus Spinning Disk IX81 microscope. Representative images are shown (scale bar = 20 µm). For each condition, 250 cells were counted. Number of VP2/VP3-expressing cells is shown as percentage, normalized to mock cells (**E**). Control HeLa cells were pre-incubated for 1 h with 0 (**F**) or 10 μM CPZ (**G**), and 5 μg/ml of Alexa Fluor 633-conjugated transferrin (Trf-A633) was added. After 15 min of incubation cells were processed for confocal fluorescence microscopy. Trf-A633 was added to CHSE-214 and cells were incubated for 1 (**H**) and 24 h (**I**). Representative images are shown (scale bar = 10 μm).

To determine the role of clathrin-mediated endocytosis in IPNV entry, we used chlorpromazine (CPZ). This cationic amphiphilic drug is known to interfere with clathrin-coated pit assembly by a reversible translocation of clathrin and its adapter proteins from the plasma membrane to intracellular vesicles^14^. CHSE-214 cells were first treated with 10 or 50 µM CPZ 1 h prior to infection and then infected with IPNV at a multiplicity of infection (MOI) of 1 PFU/cell. After extensive washing to remove unbound virus, CPZ was added at the above concentrations and maintained throughout the timecourse. As shown in Fig. 2, CPZ did not blocked IPNV infection, as the percentage of cells expressing VP2/VP3 in the presence 10 µM (Fig. 2C) or 50 µM (2D) CPZ did not differ from the mock cells (Fig. 2B). Quantification of infected cells clearly shows that CPZ treatment does not interfere with viral replication at any of the concentrations tested (Fig. 2E). As a control, we analyzed internalisation of transferrin-Alexa Fluor 633 (Trf-AF633), a well-characterized CME cargo protein^15^. After 1 h incubation, Trf-AF633 was internalised into HeLa cells (Fig. 2F) and such a process was effectively blocked by 10 µM CPZ (Fig. 2G). However, Trf-AF633- was not internalised into CHSE-214 cells neither at 1 h nor at longer incubation times (Fig. 2I). Since a validated marker for CME in CHSE-214 cells is not presently available, we cannot discard that this pathway is involved in IPNV entry.

### IPNV entry is independent of lipid raft-/caveolin-mediated endocytosis

**Figure 3:**
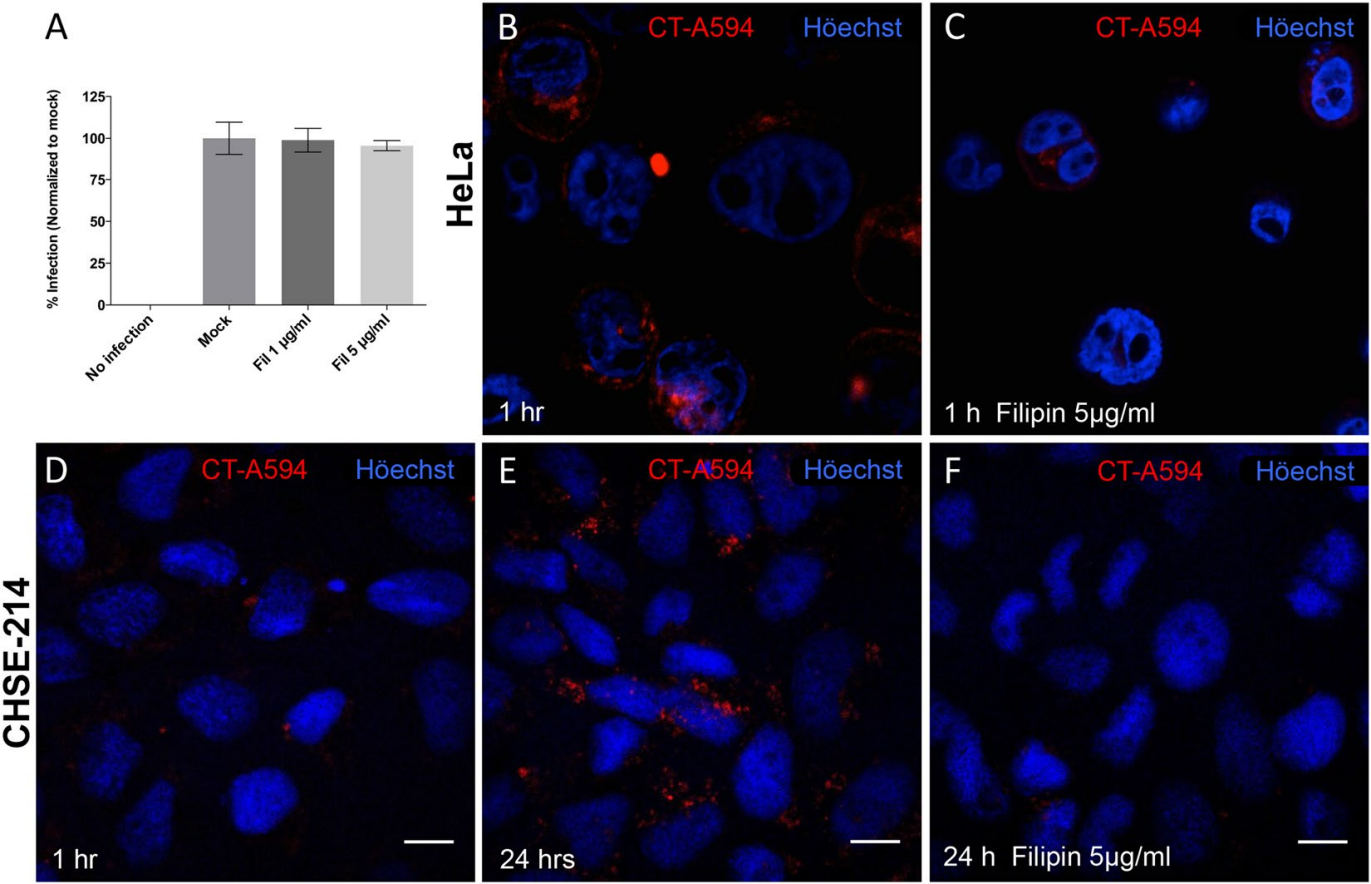
Filipin does not affect IPNV infection of CHSE-214 cells. CHSE-214 cells were infected with IPNV at an MOI of 1 for 1 h with 1 or 5 μg/ml of filipin (Fil). After 12 h of incubation at 20°, cells were processed by indirect immunofluorescence (IFI) for IPNV VP2/VP3. Images were recorded with an Olympus Spinning Disk IX81 microscope. For each condition, 250 cells were counted. Number of VP2/VP3-expressing cells is shown as a percentage, normalized to mock cells (**A**). Control HeLa cells were pre-incubated for 1 h with 0 (**B**) or 5 μg/ml (**C**) filipin and 2.5 μg/ml Alexa Fluor 594-conjugated cholera toxin B (CTB-A594) subunit were added. After 30 min, cells were processed for confocal fluorescence microscopy. CHSE-214 cells were preincubated 1 h in the absence (**D**,**E**) or with 5 μg/ml filipin (**F**). CTB-A594 (2.5 μg/ml) was added and cells were incubated for 1 h (**D**) or 24 h (**E** and **F**). Images were recorded with a C2 Plus Eclipse TI Nikon confocal microscope (scale bar = 10 µm). Representative images are shown.

To determine the role of lipid raft-/caveolin-mediated endocytosis, we used the filipin complex I, an antifungal macrolide polyene that binds to cholesterol in the plasma membrane and disrupts lipid rafts and caveolae structures^16, 17^. Cells were pretreated with 1 and 5 µg/mL of filipin for 1 h and infected with IPNV at a MOI of 1 for 1 h. After 12 h of incubation in the presence of the drug, IPNV VP2/VP3 expression was revealed by immunofluorescence. Figure 3A shows the effect of filipin on IPNV infection. No effect on viral infection was observed. Control experiments using the Alexa Fluor 594-conjugated cholera toxin B subunit (CTB-AF594) showed that a much lower amount of toxin was internalized into CHSE-214 cells (Fig. 3D) as compared to HeLa cells (Fig. 3B) after 1 h. However, at longer incubation times the amount of CTB-AF594 internalised into CHSE-214 cells increased (Fig. 3E), suggesting that lipid raft-/caveolin-mediated endocytosis is slower in CHSE-214 cells. As shown in Fig. 3F, filipin effectively reduced the levels of CTB-AF594 in CHSE-214 cells as occurs in HeLa cells (Fig. 3C). These results suggest that lipid raft-/caveolin-mediated endocytosis is active in CHSE-214 cells but it is not involved in IPNV entry.

### IPNV infection is independent of dynamin

**Figure 4:**
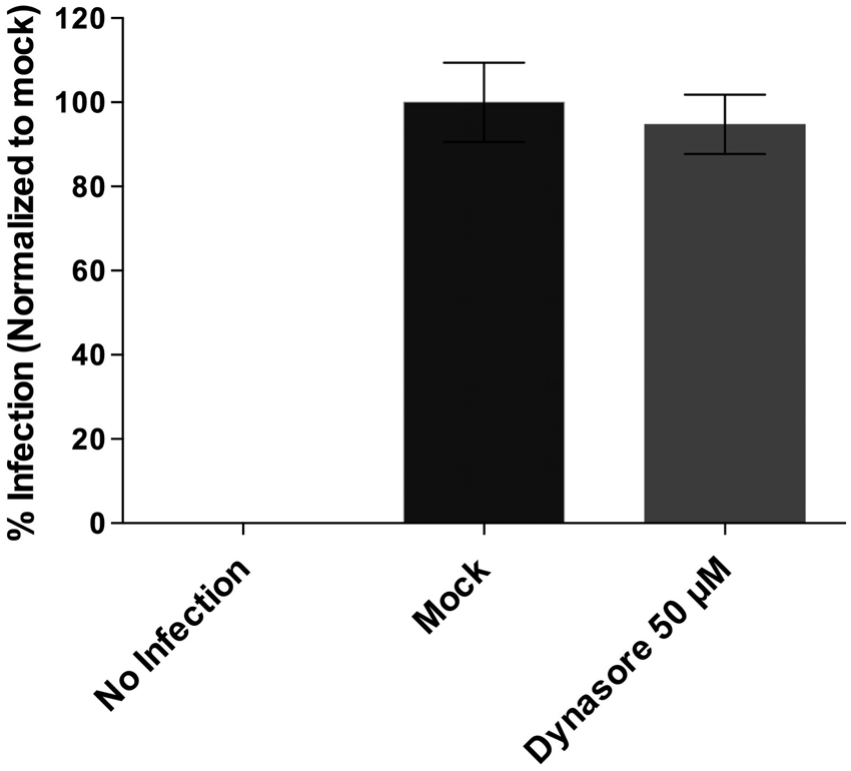
IPNV infection is independent of dynamin. CHSE-214 cells were propagated at 70–80% confluence in 24-well plates with round glass coverslips and pre incubated with 0 or 50 µM dynasore for 1 h at 20 °C. IPNV was inoculated at an MOI of 1 and led the infection proceed for 12 h at 20 °C in absence (mock) or presence of the inhibitor. Cells were processed by indirect immunofluorescence (IFI) for VP2/VP3 IPNV proteins. Imaging of fixed slides was performed with an Olympus Spinning Disk IX81 microscope, and 250 cells were counted at each condition. Number of VP2/VP3-expressing cells is shown as a percentage, normalized to mock cells.

Dynamin is a ∼100-kDa, multidomain protein belonging to a large GTPase multifamily. In addition to participating in vesicular structure formation in clathrin- and caveolin-mediated endocytosis, dynamin interacts with actin-binding proteins such as cortactin, influences the actin comet formation associated with macropinosomes and affects podosome formation, budding of transport vesicles, division of organelles and cytokinesis^18^. Classical dynamins 1, 2 and 3 are the best characterized in mammals and are found in a wide variety of species^18^. While dynamin 1 and 3 expression are restricted to specific tissues, dynamin 2 is ubiquitously expressed in mammals and has been described in fishes such as zebrafish^19^ and Salmo salar (NCBI Reference Sequence: XP_014058702.1). In order to evaluate whether IPNV entry is a dynamin-dependent process, we used the specific inhibitor dynasore. However, at 50 µM no inhibition of IPNV infection was observed (Fig. 4) suggesting that dynamin is not involved in IPNV entry.

### IPNV stimulates fluid uptake in CHSE-214 cells

**Figure 5:**
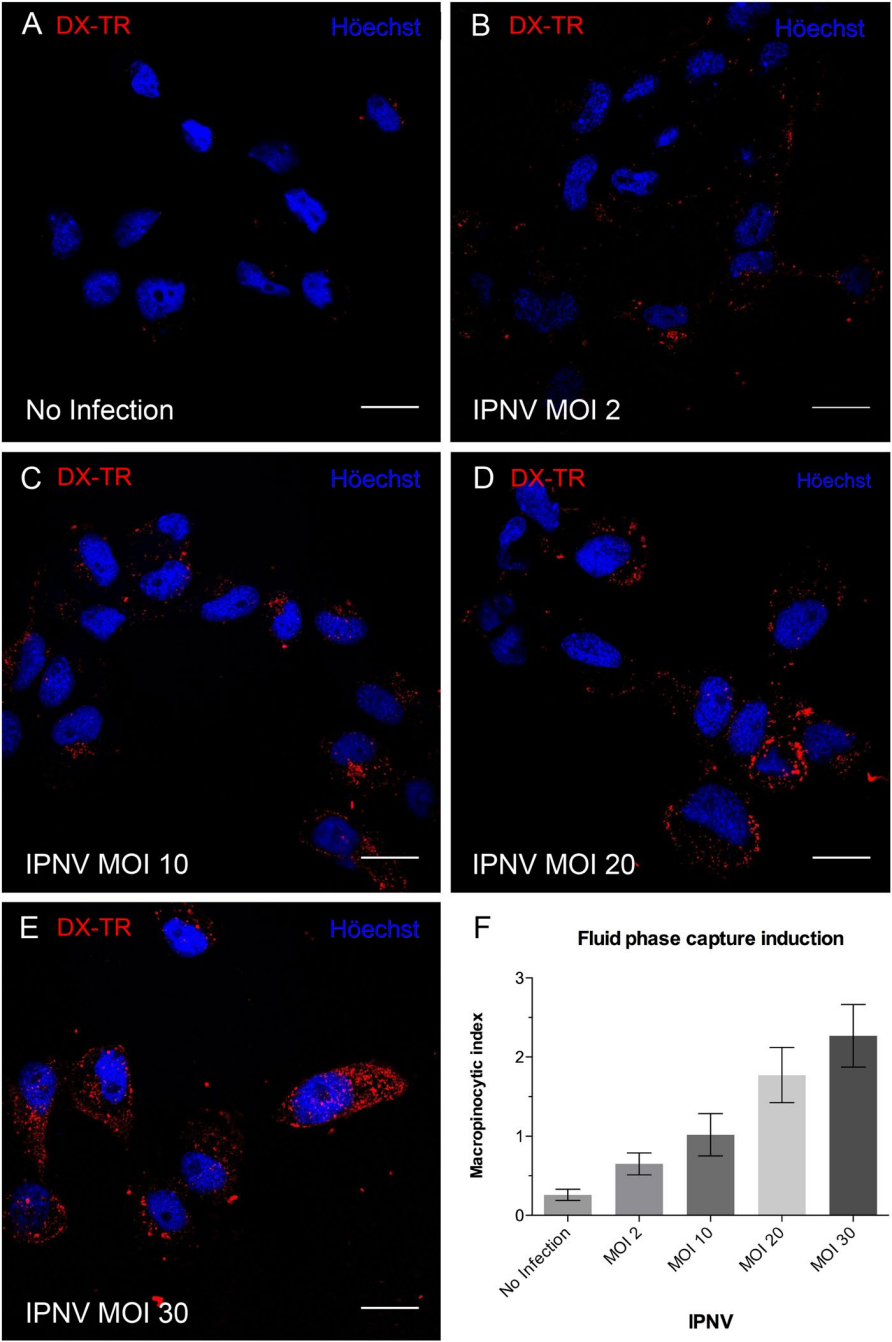
IPNV induces fluid phase capture in CHSE-214 cells. CHSE-214 cells were grown to 70% confluence in 24-well plates with round coverslips in MEM with 2% FBS. Cells were left for 16 h in MEM without FBS. IPNV at MOI of 0, 2, 10, 20 and 30 (**A**–**E**) was inoculated, then left to adsorb for 1 h at 4 °C. Dextran Texas red (250 µg/ml) was added, and cells were incubated at 20 °C for 45 min. Cells were fixed and stained with Höechst. Images were recorded with a C2 Plus Eclipse TI Nikon confocal microscope. Representative images are shown (scale bar = 10 μm). A graph of the macropinocytic index vs MOI is presented in F. The macropinocytic index was determined as described in Materials and Methods.

Macropinocytosis is an internalisation pathway used by several viruses^13^. This prompted us to evaluate whether macropinocytosis is involved in IPNV entry. A characteristic feature of macropinocytosis is extracellular fluid uptake, which may increase upon virus adsorption^20^. Thus, as a first approach, we determined the internalisation of Dextran-Texas Red® (dx-TR), a soluble fluorescent fluid-phase marker, in mock and IPNV-infected cells, to monitor macropinocytic capture. After 16 h of serum starvation, CHSE-214 cells were inoculated with IPNV. After 1 h of adsorption, dx-TR was inoculated into the culture medium and allowed to internalize for 30 min. After fixation, cells were observed with confocal microscopy to determine the macropinocytic index, as previously described^21^. Figure 5 shows that when serum starved cells were exposed to IPNV, the macropinocytic index increased linearly with the viral load (Pearson correlation coeficient = 0.99, R squared = 0.98, p = 0.001). At MOI of 30 the macropinocytic index increased about 10-fold as compared with mock cells (Fig. 5F). The substantial increase in macropinocytosis index produced by IPNV suggests that this virus is internalized by macropinocytosis.

### IPNV infection requires of actin dynamics

**Figure 6:**
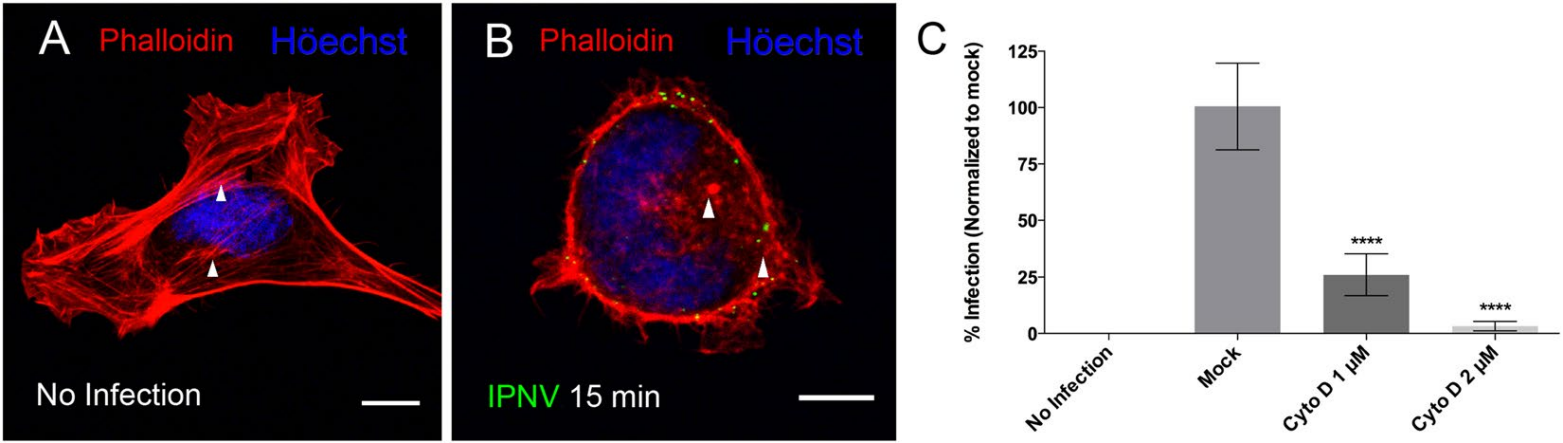
IPNV infection is dependent on actin polymerization dynamics. A. CHSE-214 cells were infected with IPNV at a MOI of 10 and adsorbed for 1 h at 4 °C. The supernatant was removed and MEM medium added at 20 °C. Cells were incubated at 20 °C for 15 min, fixed and stained with rhodamine-conjugated phalloidin. Images were recorded with a C2 Plus Nikon spectral confocal microscope, (scale = 10 μm). (**A**) no infection (**B**) 15 min post infection (white arrow heads indicate stress fibers in **A** and disorganized actin in **B**). (**C**) CHSE-214 cells were preincubated with 1 or 2 µM cytochalasin D (Cyto D) for 1 h and infected with IPNV at a MOI of 1 in the presence of Cyto D. Cells were incubated at 20 °C for 6 h and processed by indirect immunofluorescence (IFI) for IPNV VP2/VP3. For each condition, 250 cells were counted. The number of VP2/VP3-expressing cells is shown as a percentage, normalized to mock cells (****p < 0.0001).

Macropinocytosis is characterized by the formation of membrane ruffles, due to remodeling of actin filaments. The dynamics of actin polymerization and turnover involve a highly-regulated process that is crucial for macropinocytosis^13^. Given its high affinity for F-actin, rhodamine-conjugated phalloidin is frequently used to visualize actin distribution and cell morphology. Using this approach, we observed rapid changes in the distribution and structure of the actin cytoskeleton when IPNV was inoculated into CHSE-214 cells. At 15 min post infection, we observed in Fig. 6 a transition from a highly structured cytoskeleton, including highly defined stress fibers (Fig. 6A) to a disorganized structural pattern (Fig. 6B). In addition, we observed changes in cellular morphology resulting in rounded shape and smaller size. These results suggest a rearrangement of the actin cytoskeleton after IPNV virus inoculation into CHSE-214 cells, which is consistent with macropinocytosis taking place in IPNV-infected cells. Moreover we tested the effect of the actin-depolymerizing agent cytochalasin D (Cyto D) on IPNV infection. As shown in Fig. 6C, treating CHSE-214 cells with 1 or 2 µM of Cyto D reduced IPNV infection 6 hpi by about 74% or 98%, respectively, as compared to the untreated control. Treating CHSE-214 cells with Cyto D at these concentrations did not affect cell viability (not shown), suggesting that IPNV entry is an actin-dependent process.

### IPNV infection is blocked by EIPA

**Figure 7:**
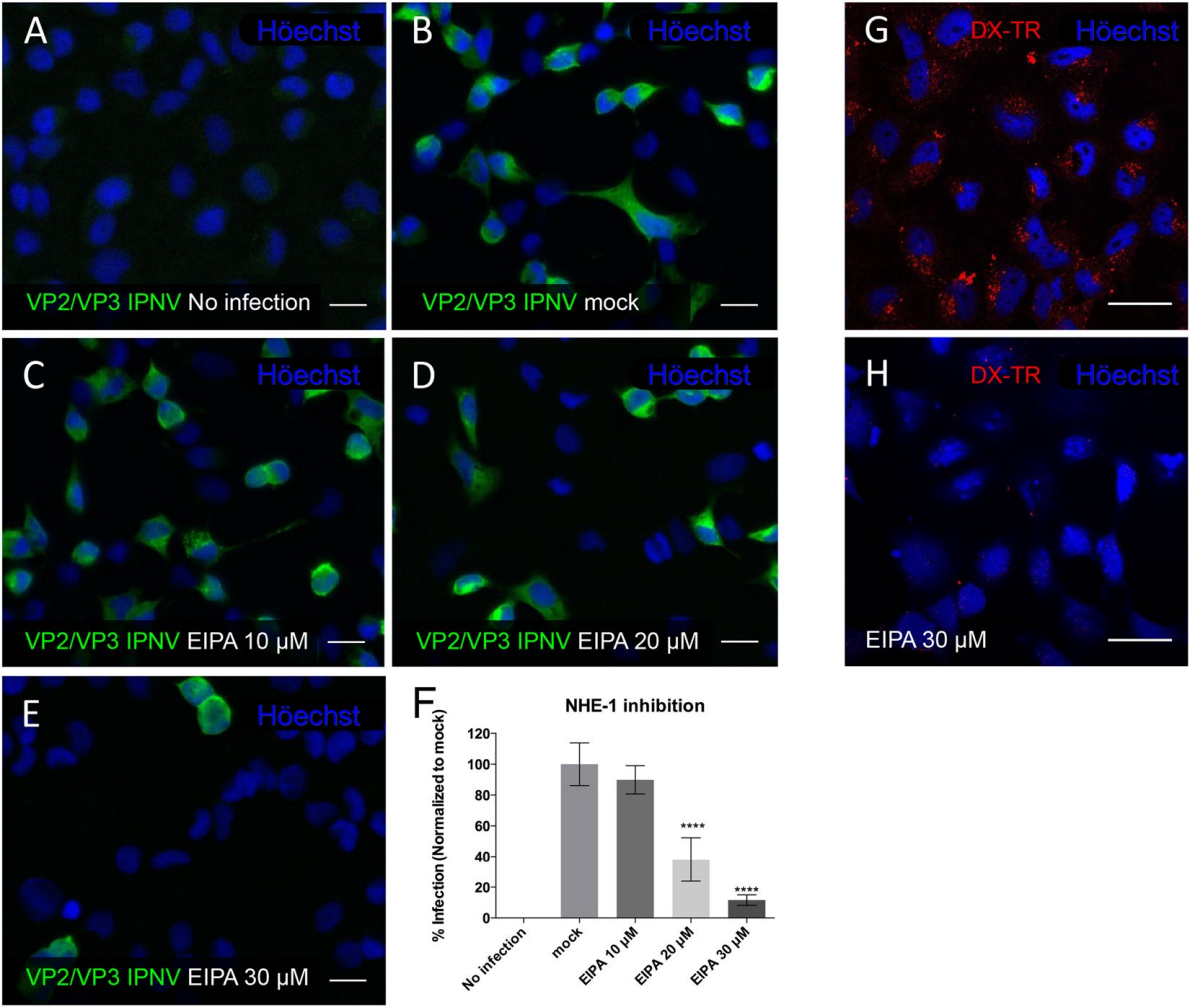
IPNV infection of CHSE-214 cells is blocked by EIPA. CHSE-214 cells were propagated to 70–80% confluence in 24-well plates with round glass coverslips and infected with IPNV at a MOI of 1 for 1 h in the presence of 0 (**B**), 10 (**C**), 20 (**D**), 30 (**E**) µM EIPA. No infection (**A**). After 12 h of incubation at 20 °C, cells were processed by indirect immunofluorescence (IFI). Representative images recorded with an Olympus Spinning Disk IX81 microscope are shown (bar scale = 20 µm). For quantification, 250 cells were counted at each condition and the number of VP2/VP3-expressing cells are shown as a percentage, normalized to mock cells (**F**) (****p < 0.0001). Control CHSE-214 cells pre-incubated for 1 h in the presence (**H**) or absence (**G**) of 30 µM EIPA and 1 mg/ml of Dx-Texas Red 70 kDa (Dx-TR) were added. After 30 min of incubation, cells were washed, fixed with 3.7% PFA-4% sucrose for 30 min and stained with Höechst solution. Images were recorded with a C2 Plus Eclipse TI Nikon confocal microscope. Representative images are shown (bar scale = 20 µm).

The NHE (Na^+^/H^+^ exchanger) is an integral membrane protein found ubiquitously in many eukaryotic organisms. This protein helps to regulate intracellular pH by exchanging intracellular protons for extracellular Na^+^ ions. NHE protects the cell from intracellular acidification and is required for cell growth and differentiation. In mammals, 9 functional NHE genes have been identified^22^. The first isoform, NHE1, is expressed in nearly all tissues^23^, whereas the other forms vary in terms of tissue and cellular distribution as well as expression level. NHE1 function has a significant impact on cytoskeletal organization through interactions with actin fibers and cell-signaling molecules^23^. Hydropathy analysis of the amino acid sequences of the 9 isoforms predicts similar topologies, although the structure of NHE1 is the best characterized (reviewed in ref. 22). NHE1 is expressed in anterior intestine, kidney and the gill of *Oncorhynchus mykiss* ^24^ and appears to have a “housekeeping” role in cell physiology. Amiloride derivatives block NHE activity, impairing the actin polymerization required for macropinocytosis^25^. As EIPA is the most effective inhibitor of NHE1^26^, we decided to use this molecule to test the role of macropinocytosis in IPNV infection. CHSE-214 cells were infected with IPNV at an MOI of 1, and VP2/VP3 peptide synthesis was measured at 12 hpi in the presence of the drug. Figure 6 shows the effect of 10, 20 and 30 µM EIPA on IPNV infection. As the concentration of EIPA increased, the percentage of infected cells decreased, suggesting that this treatment impairs IPNV entry (Fig. 7B–E). Figure 7F displays the values for these results. At 20 µM EIPA, IPNV infection decreased by more than 50%. At 30 µM EIPA IPNV infection decreased 90%. Dx-TR was used as a control to visualize macropinocytosis inhibition by EIPA (see Fig. 7G and H). When EIPA was added 1 h.pi. no inhibition of the synthesis of VP2/VP3 was observed (not shown) suggesting that the effect of EIPA occurs at an early stage of infection. These results correlate well with the suggestion that IPNV entry occurs by macropinocytosis.

### IPNV particles co-localize with Dextran-TR in intracellular compartments

**Figure 8:**
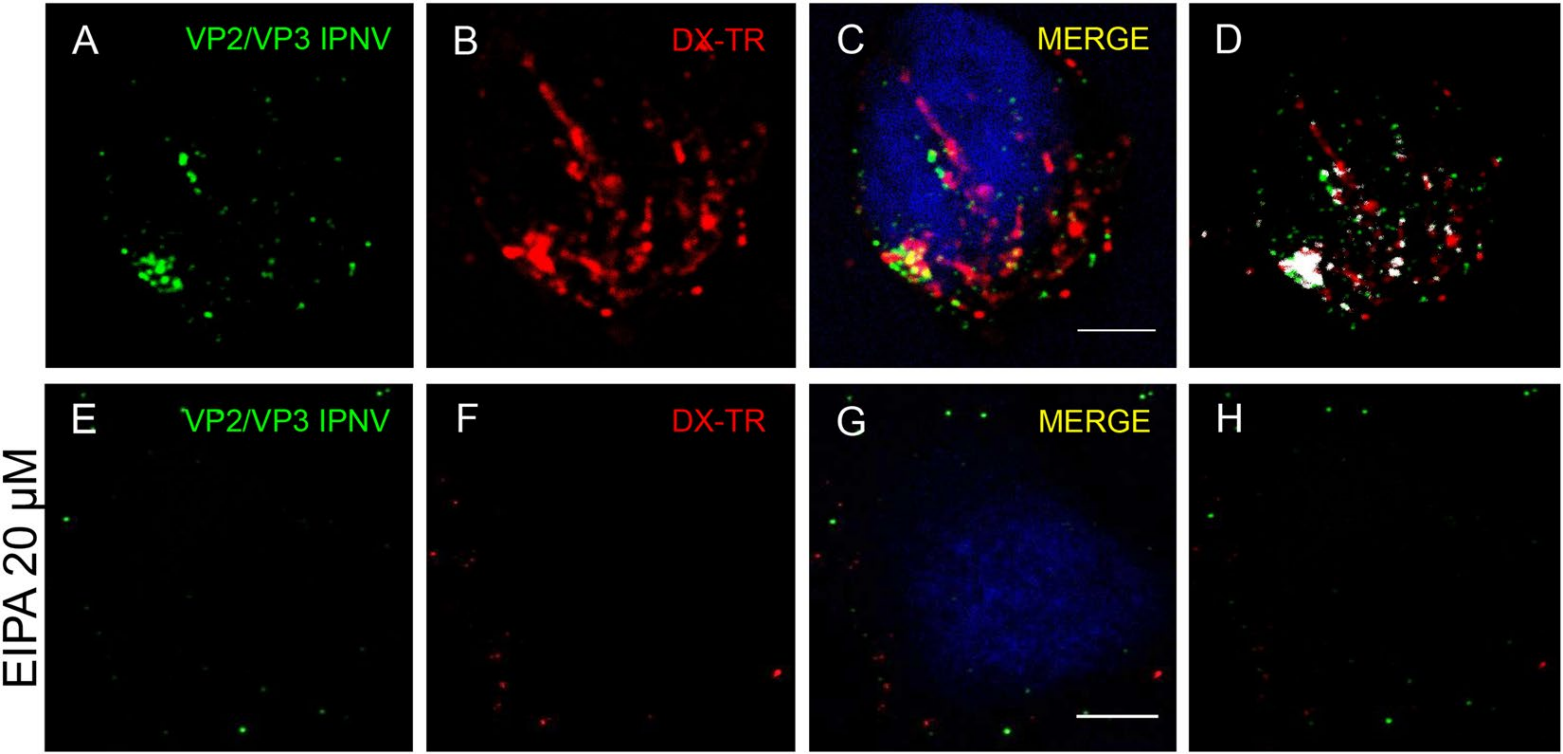
IPNV particles co-localize with Dextran-TR. IPNV at a MOI of 10 was adsorbed to serum-starved CHSE-214 cells at 4 °C for 1 h and pulsed with dx-TR for 45 min at 20 °C without (**A**–**D**) or with EIPA 20 μM (**E**–**H**). After washing, cells were processed for immunofluorescence using oligoclonal anti VP2/Vp3 IPNV antibodies and Alexa fluor®466 secondary antibody and Höechst solution for nuclear staining. Z-stacks were recorded by confocal microscopy and representative maximum Z projections are shown (scale bar = 5 µm). Analyses of co-localisation were conducted with Image J software and resulting co-localised pixel map are shown for each condition (colocalised pixels are shown as white spots) (**D** and **H**).

IPNV-induced macropinocytosis should result in the uptake of virus particles and dx-TR into macropinosomes. To determine whether IPNV particles and dx-TR co-localise, IPNV at a MOI of 10 was adsorbed to serum-starved CHSE-214 cells at 4 °C for 1 h. Then cells were pulsed with dx-TR for 45 min at 20 °C, washed, fixed and processed for immunofluorescence and laser scanning confocal microscopy. Virus particles were found in dextran-positive intracytoplasmic vacuoles (Fig. 8C). Analyses of the amount of fluorescence of the co-localizing pixels in each colour channel using Image J software, resulted in Mander split coefficient M1 of 0,39 ± 0,1571 for virus signal in the red filled pixels (M1 varies between 0 and 1, being 1 the maximum of co-localisation). In contrast, in the presence of 20 µM EIPA, a M1 coefficient of 0,0007 ± 0,0036, was obtained indicating no co-localisation (Fig. 8G) (****p < 0.0,0001).

### Role of macropinocytosis regulators during IPNV infection

**Figure 9:**
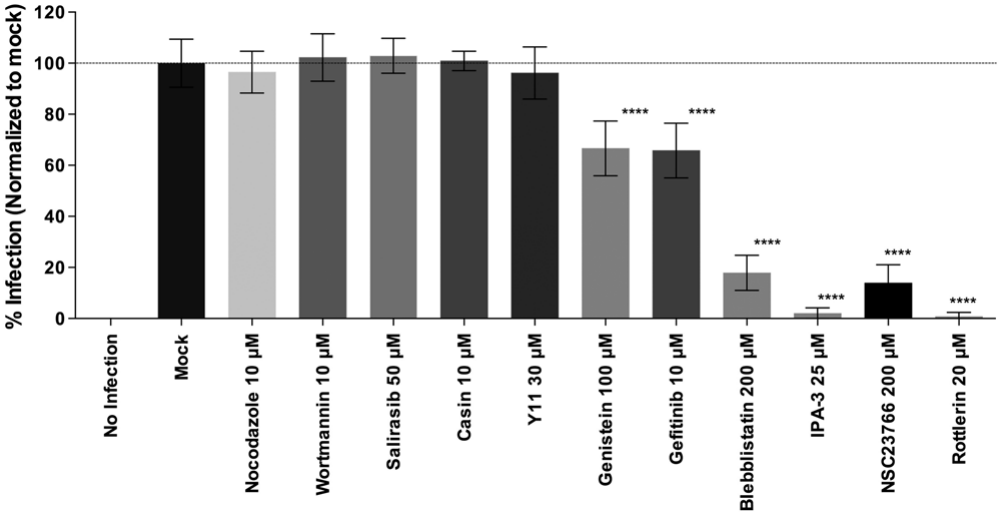
Cellular requirements for IPNV infection. IPNV infection of CHSE-214 cells was carried out in the presence of inhibitors of structural and functional cell components: nocodazole (10 μM), wortmannin (10 μM), salirasib (50 μM), casin (10 μM), Y11 (30 μM), genistein (100 μM) gefitinib (10 μM), blebbistatin (200 μM), 3-indole propionic acid (IPA-3 25 μM), NSC23766 (200 μM), rottlerin (20 μM), or no infection. CHSE-214 cells were propagated at 70–80% confluence in 24-well plates with round glass coverslips. IPNV was inoculated at an MOI of 1 for 1 h in the presence of the respective inhibitor. After 12 h of incubation at 20 °C, cells were processed by indirect immunofluorescence (IFI). Imaging of fixed slides was performed with an Olympus Spinning Disk IX81 microscope, and 250 cells were counted at each condition. Number of VP2/VP3-expressing cells is shown as a percentage, normalized to mock cells.

Several signaling pathways are involved in the actin remodeling processes that takes place during membrane ruffle formation, macropinocytic cup formation and cup closure. These pathways are triggered by activation of tyrosine kinase receptors or integrins and downstream effectors such as small GTPases, protein kinases and adaptor proteins^27^. To explore a possible role of these known regulators on IPNV infection, we used the inhibitors: wortmannin (wort) for phosfoinositol kinase 3 (PI3K), CASIN (formerly called Pirl1-related compound 2)^28^ for the cell division control protein (CDC42), farnesylthiosalicylic acid (Salirasib), that selectively disrupts the association of Ras proteins with the plasma membrane^29, 30^ and Y11 for the focal adhesion kinase (FAK). focal adhesion kinase (FAK), a downstream signal activated by integrins. As shown in Fig. 9 none of these compounds had a significant effect on IPNV infection. On the other hand, genistein (Gen), a general tyrosine kinase inhibitor, decreased IPNV infection by about 33% at 100 μM, while gefitinib (Gef), an inhibitor of epidermal growth receptor (EGFR), decreased IPNV infection by 34% at 10 μM (Fig. 9).

Rho GTPases are key regulators of cytoskeletal dynamics and are involved in many cellular processes, including migration, vesicle trafficking and cytokinesis. These proteins are conserved from plants and yeast to mammals and function by interacting with and stimulating various downstream targets, including actin nucleators, protein kinases and phospholipases. Numerous pathogens, including viruses, utilize these regulators to invade the cell^30^. Notably, NSC-23766, an inhibitor of Rac1 GTPase, reduced IPNV replication by about 86% at 200 μM (Fig. 9). Inhibition of Pak1, a downstream effector of Rac1, with 25 μM IPA-3 also decreased IPNV infection by about 98% (Fig. 9). Together, these results suggest that Rac1 could be involved in the regulation of IPNV induced macropinocytosis.

Myosin II is a motor protein powered by ATP hydrolysis, which forms an essential part of the motility machinery in most eukaryotic cells. During macropinocytosis, myosin II is regulated by Pak1 through myosin light-chain kinase (MLCK) and interacts closely with the actin cytoskeleton, providing contractile activity that can modulate ruffle movement and macropinosome closure^13, 20, 31, 32^. Myosin II can be inhibited selectively by blebbistatin (Bleb), a small molecule that is capable of binding with high affinity to the ADP-Pi-myosin complex, preventing the release of inorganic phosphate^33^. Treating CHSE-214 cells with 200 μM of Bleb reduced infection by about 82%, as compared to the untreated control (Fig. 9).

Protein kinase C (PKC) is a serine/threonine kinase activated by diacyl glycerol (DAG) and calcium as part of the signaling cascade induced by growth factors. It has been determined that PKC plays a role during macropinosome formation. In addition, PKC has been implicated in the entry processes of various viral pathogens such as rhabdoviruses, alphaviruses, poxviruses, herpesviruses and the influenza A virus^34–37^. Rottlerin (Rott) is a pharmacological inhibitor whose molecular targets are the α and δ variants of PKC, which are frequently involved in macropinocytosis^13, 38^. As observed in Fig. 9, Rott treatment nearly abolished IPNV infection at a concentration of 20 μM, suggesting a possible role for PKC in macropinocytosis.

## Discussion

Endocytosis is a general mechanism by which the cell takes up macromolecules and particles from the outside milieu, engulfing them into membranous vesicles in an energy-driven process. Endocytosis can take many forms, including clathrin-mediated endocytosis (CME), caveolin- or lipid raft-mediated endocytosis and macropinocytosis. Viruses often take advantage of these mechanisms to enter the cell and reach an optimum site for replication^12, 39^. Previous studies using electron microscopy and cationic ferritin as an endocytic tracer demonstrated that IPNV is co-internalized with ferritin into tubule-vesicular compartments in CHSE-214 cells, suggesting that endocytosis may be involved in the initial events of IPNV infection^40^. However, to date has been no evidence that might help to establish the nature of this endocytic process. The findings from the present study indicate that IPNV uses a mechanism that fulfills the major criteria used to define macropinocytosis^20^.

Macropinocytosis is constitutive in dendritic cells and macrophages but must be induced in other cell lineages^13, 39^. To engage this pathway, viruses must interact with cell-specific surface macromolecules, such as receptor tyrosine kinases (RTKs), integrin or phosphatidylserine receptor, leading to activation of downstream players which in turn produces actin rearrangements that give rise to plasma membrane protrusions or ruffles. Macropinocytic vacuoles (macropinosomes) are formed when membrane ruffles fold back onto the plasma membrane to form fluid-filled cavities that close by membrane fusion^12, 39^. Consequently, macropinosome formation is associated with a transient increase in fluid-phase uptake^20^. Although no receptor- or surface- interacting molecules for IPNV have been identified to date, we have shown that the virus induces fluid-phase capture in a dose-dependent fashion, suggesting that the virus can interact with and trigger the appropriate signaling pathway to elicit macropinocytic capture.

Cellular morphological changes associated with altered actin fiber configuration after virus inoculation suggest that significant actin dynamics are required during the initial stages of IPNV infection in CHSE-214. Accordingly, using cytochalasin D to disturb the actin cytoskeleton, we showed that IPNV infection is dependent on actin polymerization, as VP2/VP3 expression was dramatically reduced in the presence of this drug. Similar results were obtained when cytochalasin D was restricted to the first 2 hpi (data not shown), indicating that this requirement is necessary at early stages of infection, possibly at virus entry.

Due to metabolic activity during actin polymerization, large amounts of H^+^ equivalents are generated, and the sub-membranous pH is balanced by the Na^+^/H^+^ antiporter function of NHE1. Dependence on NHE1 activity is a hallmark of macropinocytosis, and its blockage induces rapid acidification of cytosol, a condition that inhibits the Rho GTPases Cdc42 and Rac1^41^. In this study, the blocking of NHE-1 by EIPA abrogated IPNV infection in a dose-dependent fashion.

Consistent with this data, we found that IPNV infection is sensible to NSC-23766 and IPA-3, suggesting dependence on the Rho GTPase Rac1 and its downstream effector p21-activated kinase (Pak1). Pak1 phosphorylates C-terminal binding protein 1 of the E1A/brefeldin A ADP ribosylated substrate (CtBP/BARS) and modulates the contractile activity of myosin II at the macropinocytic cup. Both events are critical during membrane fission and macropinosome closure^20, 31, 32, 42, 43^. Accordingly, we found that inhibition of myosin II using blebbistatin blocks IPNV infection. Myosin II is a downstream effector of Pak1^31^ suggesting a central role for Rac1 in this macropinocytic mechanism.

Globally, our results are similar to those obtained by Gimenez *et al*.^44^ in infectious bursal disease virus (IBDV). This birnavirus is associated with acute immunosuppressive disease in poultry and shares a similar amino acid sequence and capsid protein structure with IPNV^45^. The authors reported that IBDV uses macropinocytosis as a primary mechanism of cell entry. In addition, they showed that virus internalisation was significantly reduced after cholesterol depletion by MβCD, suggesting partial involvement of caveolin/lipid rafts in virus internalisation into avian cells^44, 46^. To determine whether caveolin/lipid rafts were also involved in IPNV entry, we used the polyene macrolide filipin to distort lipid rafts. Our results show that this drug does not affect the course of IPNV infection, suggesting that, unlike in IBDV, lipid rafts and caveolin are dispensable for IPNV internalisation and may not be involved in IPNV cell entry at all. In addition, control experiments using the Alexa 594-conjugated cholera toxin B subunit indicate a low activity of this pathway in CHSE-214 cells as compared to HeLa cells. However, at long incubation times we showed that the uptake of cholera toxin B subunit was effectively blocked by filipin.

Phosphatidylinositol-4,5-bisphosphate 3 kinase (PI(3)K) is an enzyme involved in various cellular functions such as cell growth, differentiation, proliferation, survival, motility and intracellular trafficking^47^. PI(3)K has also been implicated in several stages of macropinocytosis, from ruffle formation to membrane closure, as well as fusion and macropinosome traffic^13^. Surprisingly, treating CHSE-214 cells with wortmannin resulted did not inhibit IPNV infection. Given the central role that has been conferred to PI(3)K in macropinosome development, this resistance to the PI(3)K inhibitor was unexpected. However, several cases of viruses entering a cell through PI(3)K-independent macropinocytosis have been reported, including strains of the vaccinia virus (VV), human immunodeficiency virus type-1 (HIV-1) and foot-and-mouth disease virus (FMDV)^12, 48, 49^.

Clathrin-mediated endocytosis (CME) is a well-studied endocytic mechanism that is ubiquitous among eukaryotes and plays essential roles in diverse cellular functions^27, 50^. There are numerous viruses that trigger this pathway to gain access into the cell^12^. As we show in this report, treating CHSE-214 cells with chlorpromazine (CPZ), a widely-used clathrin assembly inhibitor, did not interfere with IPNV infection^14^. These observations are consistent with previous studies reporting that IPNV infection does not require low endosomal pH for cell entry^51^. IPNV infection is not dependent of GTPase dynamin activity, suggesting that the clathrin-mediated endocytic pathway would be dispensable for IPNV infection in CHSE-214 cells. However, results from the tracer for CME, transferrin, showed no internalisation into CHSE-214 cells, while it was efficiently endocytosed into HeLa cells as previously reported^27^. These data suggest that this mechanism is inefficient in our cellular model or, alternatively, the transferrin receptor does not recognize the transferrin of human origin or is absent or poorly expressed in the CHSE-214 cells. The last possibility is plausible because when Atlantic salmon head kidney cells (SHK-1 line) are inoculated with the same control, it is efficiently internalized and is blocked with CPZ treatment (data not shown). The lack of a validated tracer for CME in CHSE-214 cells does not allow us to discard CME as a pathway for IPNV internalisation.

**Figure 10:**
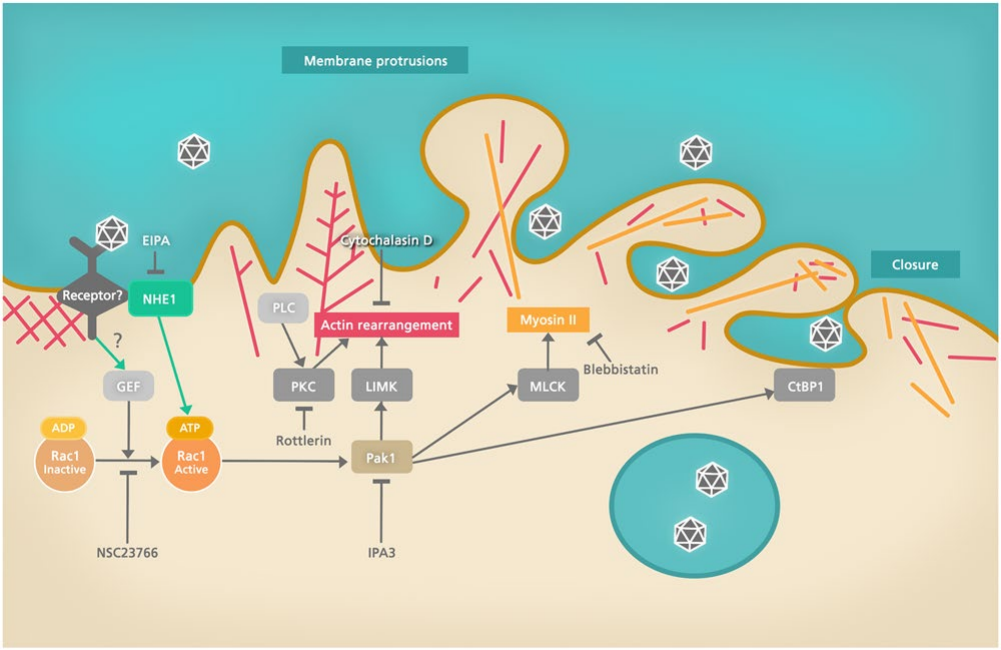
Cellular factors possibly involved in IPNV macropinocytosis. The scheme shows a commonly found factors associated to macropinocytosis pathways. Inhibition of the Rho GTPase Rac1, Pak1, PKC, myosin II and NHE1 affected IPNV infection. Inhibition sites of the drugs and efector proteins (MLCK, LIMK, CtBP1) are shown. MLCK: myosin light chain kinase, LIMK: LIM kinase, CtBP1: C-terminal binding protein 1.

In conclusion, our results suggest that IPNV enters the CHSE-214 cells by macropinocytosis. In addition, we used small inhibitor molecules as a first approach to search for potential components of the pathway involved in IPNV induced macropinocytosis. Since inhibition of EFGR, Rac1, Pak1, myosin II, and PKC (see Fig. 10) decreased IPNV infection, additional experimental research is needed to determine whether these cellular components are involved in IPNV internalisation and if so, which role they have in such process

## Materials and Methods

### Reagents

5-(N-Ethyl-N-isopropyl) amiloride (EIPA), blebbistatin, casin, chlorpromazine, cytochalasin D, dynasore, filipin complex, gefitinib (Iressa), genistein, IPA-3, nocodazole, NSC23766, rottlerin, salirasib, wortmannin, Y11, Fluoromount^TM^, polyethylene glycol 8000 and Sepharose® 6B were from Sigma-Aldrich (St. Louis, MO, USA). Alexa Fluor® 633-conjugated transferrin, Lysine fixable Dextran-Texas Red®, 70,000 MW, Alexa Fluor® 594-conjugated cholera toxin subunit B and rhodamine phalloidin were from Molecular Probes, Thermo Fisher Scientific (Waltham, MA USA).

### Antibodies and probes

Mouse anti-Vp2 mono and anti VP2/VP3 oligoclonal antibodies were obtained from Ango, Austral biologicals, (San Ramón, CA USA), Alexa Fluor® 488-conjugated donkey anti-mouse IgG, Alexa Fluor® 594-conjugated donkey anti-mouse IgG and Hoechst 3342 were from Molecular Probes, Thermo Fisher Scientific (Waltham, MA USA).

### Cells

CHSE-214 cell line (ATCC CRL-1681) derived from a Chinook Salmon (Oncorhynchus tshawytscha) embryo, were grown at 20 °C in Minimal Essential Medium Eagle (MEM) with Earle’s salts and L-glutamine (Corning, NY, USA), supplemented with 10% fetal bovine serum (FBS) (Corning, NY, USA), 100 U/ml penicillin and 100 µg/ml streptomycin. Human HeLa cells (ATCC CCL-2) were grown at 37 °C and 5% CO_2_ in the same medium supplemented with FBS and antibiotics.

### IPNV purification

CHSE-214 cells were grown at 70% confluence and inoculated with IPNV (strain Sp genogroup 5), at an MOI of 1 in a 175 cm^2^ culture flask. After 72 h, the culture supernatant was clarified by centrifugation at 5000 rpm for 10 min, aliquoted and stored at -80 °C for experiments that did not require purified virus. Otherwise, the viral particles were precipitated with 6% polyethylene glycol 8000 and 1% NaCl in PBS, pH 7.4, overnight at 4 °C, and the pellet was recovered by centrifugation at 8000 rpm for 30 min at 4 °C. Finally, the virus was purified by size-exclusion chromatography using Sepharose® 6B in PBS. The purified viral particles were titrated using end-point assay in 24-well cell culture plates^52^. The virus suspension was fractionated and stored at −80 °C.

### Immunofluorescence

CHSE-214 cells were propagated at 60–70% confluence in 24-well plates with round coverslips, 12 mm in diameter. The IPN virus was inoculated at an MOI of 1 PFU/cell^−1^ for one h and washed with PBS, pH 7.4. After 6–12 h of incubation at 20 °C, the cells were washed with PBS, fixed with 3.7% paraformaldehyde (PFA)-4% sucrose for 20 min at 37 °C, quenched with 0.1% glycine 3 min at room temperature, permeabilized with triton 0.1% for 5 min at room temperature and washed again with PBS. Mouse oligoclonal anti-VP2/VP3 antibody of IPNV (1:500) was applied for 1 h at room temperature. Excess antibody was removed by 3 washes with PBS for 5 min each and Alexa 488-conjugated donkey anti-mouse antibody (1:500 dilution) was applied and incubated for 30 min at room temperature. The coverslips were washed 3 times with PBS for 5 min, and Hoechst solution (1:1000) was applied for 15 min at room temperature, washed with PBS, rinsed with distilled water and mounted in slides using Fluoromount™. Imaging of slides was performed with an Olympus Spinning Disk IX81 epifluorescence microscope. Each condition was analyzed in triplicate, and the cells fluorescent for VP2/VP3-488 were counted in 250 cells in each slide.

### Pharmacological inhibition treatment and viral infection assays

CHSE-214 cells were grown at 60–70% confluence in 24-well plates with round coverslips, washed twice with PBS and pretreated with the appropriate inhibitor diluted to the corresponding concentration in MEM. Cells were then infected with IPNV at an MOI of 1 PFU/cell for 1 h in the presence of the corresponding inhibitor. Cells were washed with PBS, and the infection was left to proceed for additional 12 h unless a different incubation time was indicated. The inhibitors were present during the entire infection and incubation period. After incubation, cells were processed for indirect immunofluorescence analysis (IFA) and observed in an epifluorescence microscope. Each assay was performed in two independent experiments. The percentage of infected cells was determined in control and treated samples for quantification purposes using cell counter plug of Image J 1.50c4 software (NIH, USA).

### Tf, dx-TR and CTB uptake assays

Cells were seeded in coverslips to 75–80% confluence. Cells were washed 4 times with PBS, and the inhibitors EIPA, chlorpromazine or filipin complex I were added at the desired concentrations and incubated for 1 h. Cells were then washed twice with PBS. The markers Tf-633, Dx-Texas red and CTB-594 to a final concentration of 5.5 and 1 µg/mL, respectively, were added in the presence or absence of the corresponding inhibitor and cells were left at 20 °C for 15 min for Tf, 15 min for Dx-Texas red and 30 min for CTB. Cells treated with DX-Texas red were washed with 10 mM NaOAc, 5 mM NaCl, pH 5.5, and three times with PBS. Cells treated with Tf-633 and CTB were washed three times with PBS to remove free conjugates. Then cells were fixed with PFA-4% sucrose for 30 min, washed with 0.1 M glycine for 10 min. Nuclei were stained with Hoechst 1:1000 and mounted with Fluoromount™ aqueous mounting (Sigma). Cells were observed in a C2 plus eclipse TI Nikon confocal microscope. Images were processed using Image J software. The average was obtained by dividing the number of cells with band fluorescence/total number of cells × 100.

### Co-localisation analysis of IPNV particles and dx-TR

CHSE-214 were growth to 70% confluence and subjected to serum starvation for 16 hours. IPNV were inoculated at MOI of 1 and led to adsorb for 1 hour at 4 °C. The cells were pulsed with 250 μg/ml dx-TR for 45 min at 20 °C, washed four times with cold PBS, fixed with PFA-4% sucrose for 30 min and processed for IFA using anti Vp2/VP3 IPNV oligoclonal antibody followed by anti mouse Alexa fluor® 488 conjugated secondary antibody as described above. Z stacks images were acquired with a Zeiss LSM 250 laser scanning confocal microscope (100X objective). Co-localisation Mander split coefficient and significance Costes analysis were conducted with Image J 1.50c4 software using coloc-2 and colocalisation threshold plugins.

### Statistics

All experiments were performed at least in triplicate and calculated as normalized values or percentages +/− standard deviation (SD). Statistic significance test (t-test) were conducted using GraphPad Prism 6.0c software and p < 0.01 was considered statistically significant.
